# The Book World of Medicine and Science

**Published:** 1898-01-22

**Authors:** 


### THE BOOK WORLD OF MEDICINE AND SCIENCE.

INTRODUCTION TO THE STUDY OF ORGANIC CHEMISTRY. By JOHN WADE, B.Sc., London; Senior Demonstrator in the Chemical and Physical Laboratory, Guy's Hospital. Pp. 460, 8vo. Price 7s. 6d. (London: Swan Sonnenschein and Co. 1898.)

This book is, as the sub-title states, written as a text-book for students in the universities and technical schools. There is much need for such a work; the text-books in current use are more or less unsatisfactory, and the best of them—Remsen's—is scarcely adapted to English curricula. Mr. Wade's book is well printed and got up, clearly and simply written, and supplied with many diagrams and illustrations. As the author says in his preface, the book proceeds, as far as possible, from the familiar to the unfamiliar; the first part deals with the simple acids and alcohols, the second with the aliphatic, and the third with the aromatic compounds. There are two appendices; the first, at considerable length, deals with practical laboratory work, the second with qualitative analyses. A name index and a subject index complete the volume. We like the plan and execution of this book much; although examination requirements are throughout kept in view, the spectres of syllabi in nowise interfere with the author's development of his subject. Mr. Wade's book will we think find much favour with students, especially with those preparing for the scientific examinations of the University of London.

THE PRACTICE OF SURGERY. By HENRY R. WHARTON, M.D., Demonstrator of Surgery in the University of Pennsylvania; and B. FARQUHAR CURTIS, M.D., Professor of Surgery in the New York Post Graduate Medical School and the Woman's Medical College of the New York Infirmary. (London and Philadelphia: J. B. Lippincott. 1898. Price, 25s. net.)

The authors of this book recognise one of the most important facts by which the teachers of surgery are faced at the present day, namely, that the art or science of surgery has become so great, and the topics embraced by it have become so far reaching, that it is now quite impossible in the space of a single handy volume to give even a synopsis of the whole of the subject. Instead, then, of attempting the impossible, they have decided to write a surgery for the practitioner; in other words, putting on one side the wants of the pure surgeon, who always must have recourse to monographs, they have aimed at condensing within the space of one goodly volume the information which is necessary "to enable the general practitioner or the student to carry on or to begin the successful practice of the art of surgery." After a careful perusal of the twelve hundred and more pages of which this book consists, we can justly say that they have fairly and honestly performed their task. In such a work it need hardly be said that there is but little scope for brilliancy or originality. Accuracy, system, and condensation are the points to be aimed at, and there can be no doubt that these objects have been attained. The book begins most properly with a chapter on surgical bacteriology, and thus the key-note is struck at the very commencement; and when we go on to inflammation, septicæmia, pyæmia, gangrene, and other surgical diseases, instead of these conditions being treated of as entities and then explained in accordance with modern views as to bacteriology, they take their place at once as the natural and orderly results of the intrusion of micro-organisms. This is all as it should be. Again, before operations are dealt with there is a chapter devoted to asepsis and antisepsis, giving a full description of the methods of sterilisation and of the preparation of the materials used in aseptic operations. The chapter on minor surgery is a very good one, the illustrations of the various bandagings, all from photographs, being extremely well done. In following out their purpose of giving to the general practitioner a general survey of the surgical diseases of the whole body, several sections are included concerning which it is obviously impossible to give such information as can be of much use in regard to surgical treatment. The section on diseases of the eye, for example, contains details regarding operations for cataract which can only be taken as indicating the general direction which treatment in the hands of a specialist may be expected to take. They certainly must not be accepted as suggesting that the general practitioner is to try his hand at cataract extraction. On the other hand, we think that in regard to diseases of the ear, which often involve emergencies in which the practitioner ought to be able to intervene without delay, many more details might have been given than are to be found. Nevertheless, the book, taken as a whole, will be found to be a very useful one, and as a general practitioner's guide will occupy a high place. It is well and profusely illustrated. Many of the woodcuts are old friends, but a considerable number are new, and it may be said generally that they are used with judgment and not for mere display. If we were to criticise them at all we would suggest that some of them appear to be taken from extreme cases rather than from such as are likely to be commonly met with, and that in some cases, such as one of extreme œdema of the arm from carcinoma of the breast, the special vices of photography have been used to exaggerate the deformity; still even these serve to illustrate the points spoken of in the text, and that, after all, is the use of such pictures so far as teaching is concerned.

PSILOSIS OR "SPRUE": ITS NATURE AND TREATMENT. By GEORGE THIN, M.D. Second aud enlarged edition. (London: J. and A. Churchill. 1897.)

This is a carefully written monograph on a subject of great interest to all who practise in the East, or have much to do with those who have dwelt in India, China, or the Eastern Archipelago. Psilosis is one of the three diseases—malarial fever and dysentery being the other two—which are the chief agents in collecting the blood-tax which the maintenance of our commerce and our Eastern possessions entails upon this country, and it is a disease which is responsible for much ill-health among people who have returned to Europe after a residence in the East. The leading feature of psilosis is a rawness or bareness of the tongue and intestinal mucous membrane, and it may be briefly described as a chronic affection of the intestine, usually associated with irregularity or looseness of the bowels, localised inflammatory lesions of the tongue and buccal mucous membrane, and tenderness of the gullet. There is usually no blood or mucus in the stools and no straining, the motions being of a very pale straw or yellowish colour, unformed and frothy. It is an interesting question whether the "sprew" of the Dutch physicians of Java, the Cochin China diarrhœa of the French, and the white diarrhœa or hill diarrhœa of the Indian writers, is one and the same disease; and into this question Dr. Thin enters very fully, coming to the conclusion that the Dutch sprew is a separate and distinct disease. Some cases are recorded with great thoroughness, and the overwhelming importance of milk diet in the treatment of the disease is strongly insisted on. From a clinical point of view, the most interesting parts of the book are those which deal with the symptoms of the disease and those which are devoted to a description of the minutiæ which must be observed in the administration of a milk diet if success is to be achieved. It is a book well worth perusal.

